# Stimulation dependent induction of fear and depression in deep brain stimulation: a case report

**DOI:** 10.4076/1752-1947-3-9136

**Published:** 2009-09-11

**Authors:** Michael Sabolek, Ingo Uttner, Klaus Seitz, Eduard Kraft, Alexander Storch

**Affiliations:** 1 Department of Neurology, EMA-University of Greifswald, Greifswald, Germany; 2 Department of Neurology, University of Ulm, Ulm, Germany; 3 Department of Neurosurgery, University of Ulm, Günzburg, Germany; 4 Department of Physical Medicine and Rehabilitation, Clinic Großhadern, University of Munich, Munich, Germany; 5 Department of Neurology, Dresden University of Technology, Dresden, Germany

## Abstract

**Introduction:**

Psychiatric side effects of deep brain stimulation are not uncommon. It is often limited to transient mood alterations. We report for the first time a case of acute stimulation-dependent fear during intraoperative test stimulation.

**Case presentation:**

During test stimulation for electrode placement to the left subthalamic nucleus, a 58-year-old caucasian man with Parkinson's disease developed a severe reproducible feeling of fear together with elevated heart rate and sweating. Postoperatively, the patient developed a therapy refractory major depression in spite of excellent motor-control. Reprogramming the stimulator using a more rostral contact resulted in an abrupt and complete disappearance of the depressive syndrome.

**Conclusion:**

Postoperative re-evaluation of the stimulation site of the patient inducing acute fear by analyzing his intraoperative microrecordings and Talairach coordinates revealed stimulation within his right substantia nigra. The contrast analysis of the postoperative stimulation site suggests induction of depression in the patient by stimulation of the caudal part of his subthalamic nucleus. Acute psychiatric side effects of deep brain stimulation are relatively rare but must not be overlooked while concentrating on the improvement of motor deficit.

## Introduction

Bilateral deep brain stimulation (DBS) in the subthalamic nucleus (STN) is an accepted and standardized therapy in patients of advanced Parkinson's disease (PD) [1]. Permanent STN-DBS leads to an average of 50% improvement of motor function [2] and allows for the reduction of antiparkinsonian medication to approximately 50-65% of the pre-operative dosage [3]. It is well accepted that chronic STN-DBS not only affects motor function of patients, but also their psychic behaviour, including impairment of their executive functions and cognition as well as mood changes like mania and depression [4]-[9]. There are, however, very few reports of acute, stimulation dependent mood changes among patients [9]-[11]. Here we report the first case of acute severe stimulation-dependent fear.

## Case presentation

A 58-year-old Caucasian man with a 13-year disease history of Parkinson's disease was experiencing severe motor fluctuations. His preoperative medication included high doses of pergolide (24 mg/d) and levodopa (1400 mg/d) plus entacapone. The decision was made to implant bilateral DBS electrodes into the STN of the patient. Preoperatively, there were no signs of anxiety or depression (Beck-Depression-Inventory: 3). Implantation trajectories and target points were planned using stereotactic CCT (cerebral computed tomography) technology and FrameLink™ stereotactic planning software. The calculated STN positions (Table 1) were in the normal range of STN positions reported in the medical literature [12]-[14]. Intraoperatively the electrode positions were adjusted using a Leksell® stereotactic arc. Intraoperative neurophysiological recordings were performed using a five microelectrode recording system (LeadPoint®, Medtronic Inc.). During test macro-stimulation (right hemisphere), 3 mm below the calculated target point (Table 1), the patient experienced sudden severe fear together with sudden elevation of blood pressure [> 210 mm Hg systolic], tachycardia [> 150/min.], tachypnoea and severe sweating, which was already at a current of 1.5 mA. After terminating the stimulation, the fear completely vanished in a few seconds. The phenomenon was reproducible in a second unannounced test-stimulation. Another test stimulation, 2 mm more rostral, provided excellent motor symptom control with no apparent side effects, so the DBS electrodes were implanted in this position. Postoperative physical recovery was promising (Table 1). However, the patient constantly complained of feelings of sadness, depression, diffuse anxiety, reduced drive and loss of interest. The clinical picture met the criteria for a major depression according to DSM IV and ICD-10. Ratings of *Hamilton Depression Scale* (HAMDS) and *Beck Depression Inventory* (BDI) were also compatible with the clinical diagnosis of major depression (Table 1). Standard treatment with selective serotonin reuptake inhibitors (SSRI) had no effect. Extensive neuropsychological examination (memory [block and word span, Munich Verbal Memory Test, Continuous Visual Memory Test, Boston Naming], attention [Trail Making Test, Stroop Test], frontal executive functions [Controlled Oral Word Association Test, Semantic Fluency, Colored Progressive Matrices] and intelligence [Vocabulary Test]) revealed no substantial cognitive impairment. Since persistent treatment-resistant postoperative depression is unusual [5,6] after 3 months we decided to try to change the stimulation parameters despite excellent motor control. After terminating the stimulation, severe bradykinesia and tremor reappeared within seconds. Nevertheless, the patient reported a fast and pronounced improvement of mood which correlated with HMDS and BDI scoring (Table 1). With the patient's consent, we tested the reproducibility of the induction of depression and found acute onset of depression and diffuse anxiety after restarting the stimulation. Afterwards, we altered the stimulation parameters using a more rostral contact on both sides (Table 1). These parameters provided good motor control with no side effects on mood. BDI and HAMDS remained normal (Table 1). At all points, standard stimulation parameters (130 Hz, 60 µs, up to 3.5 V) were used.

**Table 1:** Electrode positions and clinical stimulation effects

Position in relation to mid-commissural point	Left			Right			UPDRS III	BDI	HAMDS
	x (medial-lateral)	y (ventral-dorsal)	z (rostral-caudal)	x (medial-lateral)	y (ventral-dorsal)	z (rostral-caudal)						
Calculated STN position	−12	−2	−4	12	−2	−4			
Intraoperative macrostimulation	−9	−2	−8	**11**	**−2**	**−7**			
Postoperative stimulation							45	4	5
Stimulator off									
Contact position:depression	−9	−2	−4.5 to −3.0	11	−3	−3.5 to −2.0	12	14	14
Contact position: no depression	−9	−2	−2.5 to −1.0	11	−3	−1.5 to 0.0	14	3	5

STN: Subthalamic nucleus.

BDI: Beck depression inventory.

HAMDS: Hamilton Depression scale.

UPDRS III: Unified Parkinson's disease rating scale, motorscale.

Bold Boxes: Fear induction at this electrode position.

## Conclusion

While STN-DBS in advanced PD patients leads to impressive improvement in motor disability, several reports mention possible psychiatric side effects [6]-[9]. Whereas depressive episodes are reported in a subgroup of patients [11], most patients experience a long-term improvement in depression scores in comparison with preoperative state. Long lasting depressive state is an uncommon side effect of STN stimulation [5,11]. Other symptoms such as lack of initiation, apathy, social withdrawal, lability, moodiness, insensitivity and mania were also reported [5,9,11,15]. In a recent article, apathy, defined as *"lack of motivation, interest or emotions,"* was attributed to a direct stimulation of the STN [15]. These symptoms seemed apparent in our patient. Therefore, this known side effect may have contributed to the clinical picture interpreted as depressive mood. In contrast to these chronic side effects, there are few reports about an acute influence of DBS on mood [9]-[11]. This is the first report of acute stimulation-dependent induction of fear. Here the electrode position was 3 mm caudal of the calculated target point for the STN and, according to Talairach coordinates, within the substantia nigra pars reticulate (SNR) (Table 1). Re-evaluation of the intraoperative micro-recordings confirmed that the stimulation position was 0.5 mm caudal of the beginning of SNR typical signals. The electrode position inducting the postoperative depressive state, re-evaluated using postoperative stereotactic CCT technique, was 3.5 mm rostral of this position and clearly away from the SNR. On both sides it was very close to the calculated STN position (Table 1) and at the lower end of STN typical signals during intraoperative micro-recording. In a prospective study of 17 patients, intraoperative stimulation-dependent autonomic side effects were reported in 19.6% of the test stimulations at a mean voltage of 3.1 V. The nature of these autonomic side effects was heterogeneous (confusion, malaise, chest congestion, abdominal discomfort, feeling of anguish, anxiety or stress, feeling of warmth or cold that was either diffused or restricted to the face, unilateral or bilateral mydriasis, diffused or local excessive sweating, diffused or hemifacial flushing, mild tachycardia and mild hypertension) [16]. These side effects were observed during stimulation in an area of 3.2 × 4.6 × 2.4 mm and the calculated mean point was within the STN according to Talairach coordinates [16]. Given the mean diameter of the STN of 2.5-2.5 mm and the variety of side effects, a direct attribution of all of these side effects to one particular spot is difficult. Also the authors of this article expressed concern that the stimulation volume during a stimulation of 3.1 V may be sufficient to involve autonomic structures neighbouring the STN [16]. In our case, the direct induction of fear was observed at very low currents of 1.5 mA. Therefore, unintentional stimulation of neighbouring structures due to a large electrical field is unlikely. This strongly suggests that the observed induction of fear was due to direct stimulation of the SNR. Furthermore, this side effect appeared at the position, where SNR typical signals were recorded prior to test-stimulation. Our findings may contribute to intraoperative electrode localisation particularly in cases where intraoperative micro-recording shows no clear SNR signals. It means that the appearance of acute fear during test stimulation might indicate an electrode position within the SNR.

## Consent

Written informed consent was obtained from the patient's children for publication of this case report and accompanying images. This was necessary as the patient himself subsequently died. A copy of the written consent is available for review by the Editor-in-Chief of this journal.

## Competing interests

The authors declare that they have no competing interests.

## Authors' contributions

MS and AS performed intraoperative micro-recording, macro-stimulation and functional testing. KS performed the surgical procedure and the trajectory planning. EK and KS performed the postoperative interpretation of the different stimulation sites concerning Talairach coordinates. MS and AS performed the postoperative interpretation of the micro-recording results. MS and IU performed postoperative video documentation during reprogramming. IU performed all pre- and postoperative neuropsychological examinations and tests and interpreted these. MS and AS finalized the manuscript.
